# Handgrip strength predicts length of hospital stay in an abdominal surgical setting: the role of frailty beyond age

**DOI:** 10.1007/s40520-022-02121-z

**Published:** 2022-04-07

**Authors:** Luigi Marano, Ludovico Carbone, Gianmario Edoardo Poto, Margherita Gambelli, Leonelle Lore Nguefack Noudem, Giulia Grassi, Fabiana Manasci, Giulia Curreri, Alessandra Giuliani, Riccardo Piagnerelli, Vinno Savelli, Daniele Marrelli, Franco Roviello, Virginia Boccardi

**Affiliations:** 1 Unit of General Surgery and Surgical Oncology, Department of Medicine Surgery and Neurosciences, University of Siena, Siena, Italy; 2 Section of Gerontology and Geriatrics, Department of Medicine and Surgery, University of Perugia, Perugia, Italy

**Keywords:** Handgrip strength, Hospital stay, General surgery, Frailty, Prognosis

## Abstract

**Background:**

Chronological age per se cannot be considered a prognostic risk factor for outcomes after elective surgery, whereas frailty could be. A simple and easy-to-get marker for frailty, such as handgrip strength (HGS), may support the surgeon in decision for an adequate healthcare plan.

**Aims:**

The aims of this study were to: (1) determine the prevalence of frailty in an abdominal surgery setting independent of age; (2) evaluate the predictive validity of HGS for the length of hospital stay (LOS).

**Methods:**

This is a retrospective study conducted in subjects who underwent abdominal surgical procedures. Only subjects with complete cognitive, functional, nutritional assessments and available measurement of HGS at admission were included. A final cohort of 108 patients were enrolled in the study.

**Results:**

Subjects had a mean age of 67.8 ± 15.8 years (age range 19–93 years old) and were mostly men. According to Fried’s criteria, 17 (15.7%, 4F/13 M) were fit, 58 (23.7%; 24F/34 M) were pre-frail and 33 (30.6%; 20F/13 M) were frail. As expected, HGS significantly differed between groups having frail lower values as compared with pre-frail and fit persons (fit: 32.99 ± 10.34 kg; pre-frail: 27.49 ± 10.35 kg; frail: 15.96 ± 9.52 kg, *p* < 0.0001). A final regression analysis showed that HGS was significantly and inversely associated with LOS (*p* = 0.020) independent of multiple covariates, including age.

**Discussion:**

Most of the population undergoing abdominal surgery is pre-frail or frail. The measurement of handgrip strength is simple and inexpensive, and provides prognostic information for surgical outcomes. Muscle strength, as measured by handgrip dynamometry, is a strong predictor of LOS in a surgical setting.

## Introduction

The number of surgical procedures in adult and older populations has increased in the past few decades [1–3]. Recent studies have clearly shown that in elderly, age itself is not a prognostic risk factor for complications after elective surgery, including length of hospital stay (LOS), whereas functional frailty is [4]. Frailty is a complex, multidimensional, and cyclical state of diminished physiologic reserve that results in decreased resilience and adaptive capacity of a person, and it is characterized by increased vulnerability to many stressors. The concept of frailty has become increasingly recognized as the most important status for health outcomes, with particular interest in subjects who are undergoing major surgery. Interestingly, it has been demonstrated that frail older patients are at increased risk of postoperative complications as well as LOS [5, 6]. However, frailty is often misunderstood in surgical settings, not identified and confused as a simply hallmark of aging. Thus, surgical patients are usually assessed by age, while frailty, independent of chronological age is still undervalued in such a setting. Interestingly, a recent study showed that frailty—using the Accumulation of Deficits and Fried models—is prevalent even in younger adults (18–65 years old), and its prevalence varies depending on which frailty tool is used [7]. Accordingly, a more recent study found that frailty—assessed by completion of the Canadian Study of Health and Ageing—exists in adults admitted as surgical emergency [8], while no evidence is available in abdominal surgery setting to adverse outcomes.

Collectively, an easier to-get marker of frailty could be the key for immediate clinical use in the surgical setting beyond age. In this context, handgrip strength (HGS) is a useful marker of frailty. HGS is a practical and objective measure of overall muscle strength [9] functions as well as a component of comprehensive geriatric assessment  [10–13]. However, its use within surgical settings has not been rigorously validated. Age and gender were described as the strongest factors influencing HGS in healthy subjects. Accordingly, HGS declines with increasing age [14] with lower values for women [15]. Interestingly, HGS has been shown to predict LOS among some surgical [16, 17] and cancer patients [18], but it is unknown as to whether a similar association exists in abdominal surgery setting. Interestingly, LOS is an important health-care outcome of interest due to the resource intensiveness of a hospital bed. Although less well studied, there is a growing body of literature investigating the value of frailty specifically in oncologic surgery [19, 20]; however, no study has focused on the HGS value. Considering such evidence, the aims of this study were to: (1) determine the prevalence of frailty in general surgery and surgical oncology setting independent of age; (2) evaluate the predictive validity of HGS for the LOS and its relationship with frailty as a whole. A simple and easy to get marker, such as HGS, potentially predicting LOS, may be useful as an important task to support the decision of an adequate health-care plan by the medical team and for an efficient management of hospital resources.

## Methods

### Subjects and study design

This a retrospective study conducted in adult and older people who underwent abdominal surgical procedure for non-oncological (general surgery) and oncological (general oncological surgery) diseases between July 2020 and August 2021 at Department of Surgery of the University of Siena. Only patients operated in elective setting, with available measurement of handgrip at admission and who were able to give a written informed consent, were included. Data on demographics, anthropometrics, physical examination and clinical information were gathered from the hospitalization chart. We followed the Strengthening the Reporting of Observational Studies in Epidemiology (STROBE) reporting guideline. This study was approved by the Internal Institutional Review Board.

### Groups definition

Frailty is typically assessed in older populations. Identifying frailty in adults aged under 65 years may have crucial value, if it supports the delivery of timely care as in a surgical setting. In populations that included people aged over and under 60 years, Fried frailty phenotype demonstrated predictive validity [21]. Thus, subjects were divided into three groups according to Fried’s criteria for frailty [22]. Fried’s phenotype method classifies older adults as fit, pre-frail or frail based on five criteria: weight loss, exhaustion, low physical activity, slowness, and weakness. The stages of frailty, based on Fried criteria, were defined as follows: a score of 0 means that a person is fit or not frail. People with a score of 1 or 2 are at intermediate risk for adverse outcomes or are considered to be pre-frail. A score of 3–5 indicates that someone is frail [22].

### Analytical method

Weight and height were measured by standard technique. Body mass index (BMI) was calculated as weight in kilograms divided by square of height expressed in meters.

### Cognitive, functional, and nutritional assessment

Cognitive performances were evaluated with the Mini-Mental State Examination (MMSE) as a test of general cognition [23]. To avoid the underestimation of a self-rated level of functional capacity, an informant-based rating of functional status was carried out using the Basic Activities of Daily Living (BADL) [24] and the Instrumental Activities of Daily Living (IADL) scales [25]. BADL includes six activities: bathing, dressing, toileting, transferring, continence, and feeding. IADL includes eight activities: using the telephone, shopping, meal preparation, housekeeping, laundry, use of transportation, self-administration of drugs, and handling finances. Any dysfunction in the performance of these activities was recorded as dependence in the correspondent item. BADL score ranges from 6 (total independence) to 0 (total dependence), and IADL from 8 (total independence) to 0 (total dependence). The nutritional status was assessed by the administration of the Mini Nutritional Assessment (MNA) [26]. The MNA has been developed to assess malnutrition in old age subjects and to select those who might get benefited from early diagnosis and treatment. It is completed by a medical doctor and comprised 18 questions on: (1) anthropometry; (2) questions on dietary intake and habits; (3) general assessment; and (4) self-assessment. After completing the whole questionnaire, the total score (a maximum of 15 points) allows grouping the nutritional status according to clearly defined verges: scores above 12 are defined as good status; scores 8–11 mean at risk of malnutrition; scores below 7 are defined as malnourished [26].

### Handgrip strength assessment

Maximal isometric handgrip strength was measured with a portable adjustable handgrip dynamometer (Deyard EH101). The handgrip was measured in kilograms (kg). The participants adopted a seated, upright position, shoulders abducted and neutrally rotated, elbow extended, the forearm in neutral position, and the wrist at extension between 0° and 30°. Three maximal voluntary contractions, each with a 5-s duration, were performed for each hand, alternating between the right and left hands to avoid muscle fatigue. The highest value for the right and left hand were used in the analysis.

### Statistical analysis

The observed data were normally distributed (Shapiro–Wilk *W* test) and are presented as means ± standard deviation (SD). To assess differences among groups, ANOVA or Pearson’s Chi-squared (*χ*^2^) test was used, as appropriate. Simple and partial (controlling for age and gender) correlations were used to test relations between HGS and LOS. The independent effect of HGS on LOS (dependent variable) was tested by a linear regression controlling by multiple covariates, including age, gender, BMI, type of surgery, and MNA. Sample size calculation was estimated by GPower 3.1.7 software (http://www.softpedia.com). The resulting total sample size, estimated according to a global effect size of 40% with type I error of 0.05 and a power of 96%, was 105 subjects. All *p* values presented are two tailed; a value of *p* ≤ 0.05 was considered significant. All *p* values are two tailed, and the level of significance was set at *p* ≤ 0.05. Statistical analyses were performed using the SPSS 20 software package (SPSS, Inc., Chicago, IL).

## Results

### Sample characteristics

A total of 130 subjects were selected. Among these, 12 refused to participate and 10 were unable to give their consent, leaving a cohort of 108 subjects eligible for the study. Table 1 shows the demographic and clinical characteristics of the whole cohort. Subjects had a mean age of 67.8 ± 15.8 years (age range 19–93 years old) and were slightly overweight. Forty-six patients (42.6%) underwent general surgery and 62 (57.4%) general oncological surgery. Table 2 shows the demographic and clinical characteristics of the whole cohort stratified by type of surgery. Subjects who underwent general oncological surgery had a significant lower score in MNA (mostly at risk of malnutrition, *p* = 0.004) and a longer LOS (16.2 ± 12.0 vs 8.0 ± 9.9; *p* < 0.001) as compared with patients who underwent general surgery.

**Table 1:** Population sample characteristics (*n* = 108)

Age (years)	67.8 ± 15.8
M/F (*n*)	60/ 48
BMI (kg/m^2^)	25.9 ± 4.6
BADL	5.40 ± 1.56
IADL	6.33 ± 2.17
MMSE	25.09 ± 6.76
MNA	9.59 ± 3.13
LOS (days)	12.7 ± 11.8

Unless otherwise noted, data are presented as means ± SD

*BMI* body mass index, *M* male, *F* female, *BADL* Basic Activities of Daily Living, *IADL* Instrumental Activities of Daily Living, *MMSE* Mini-Mental State Examination (corrected by age and education), *MNA* Mini Nutritional Assessment, *LOS* length of hospital stay

**Table 2:** Clinical characteristics of subjects stratified by the presence of cancer (*n* = 108)

	General surgery	General oncological surgery	*p*
*N* (%)	46 (42.6)	62 (57.4)	
Age, years	65.2 ± 18.5	69.7 ± 13.2	0.148
Gender F/M, *n*	19/27	29/33	0.356*
BMI	25.6 ± 5.4	26.2 ± 4.6	0.515
BADL	5.59 ± 1.52	5.26 ± 1.58	0.282
IADL	6.63 ± 2.01	6.11 ± 2.27	0.223
MMSE	26.1 ± 6.0	24.3 ± 7.1	0.155
MNA	10.5 ± 3.1	8.8 ± 2.9	0.004
LOS	8.0 ± 9.9	16.2 ± 12.0	< 0.0001

Unless otherwise noted, data are presented as means ± SD

*BMI* body mass index, *M* male, *F* female, *BADL* Basic Activities of Daily Living, *IADL* Instrumental Activities of Daily Living, *MMSE* Mini-Mental State Examination (corrected by age and education), *MNA* Mini Nutritional Assessment, *LOS* length of hospital stay

*χ^2^ = 0.320

### Sample characteristics stratified by frailty status

According to Fried’s criteria 17 patients (15.7%, 4F/13 M) were fit, 58 (23.7%; 24F/34 M) were pre-frail and 33 (30.6%; 20F/13 M) were frail. Women were more likely to be frail (χ^2^ = 7.723, *p* = 0.035). Table 3 shows the demographic and clinical characteristics of the whole cohort stratified by frailty status. Frail subjects were older and with lower scores in BADL, IADL, MMSE and MNA. No difference was found between groups in the LOS even if a trend was found in a post hoc analysis between fit and frail (*p* = 0.08). No difference was found between groups in the type of surgery (*χ*^2^ = 4.053; *p* = 0.130, data not shown).

**Table 3:** Clinical characteristics of subjects stratified by frailty status (*n* = 108)

	Fit (0)	Pre-frail (1–2)	Frail (≥ 3)	*p*
*N* (%)	17 (15.7)	58 (53.7)	33 (30.6)	
Age, years	58.4 ± 14.7	63.8 ± 14.6	79.7 ± 11.2	< 0.0001
Gender F/M, *n*	26/47	26/47	21/13	0.035*
BMI	25.2 ± 4.8	26.1 ± 4.6	26.1 ± 4.6	0.751
BADL	5.88 ± 0.33	5.93 ± 0.69	4.21 ± 2.27	< 0.0001
IADL	7.82 ± 0.52	7.05 ± 1.08	4.30 ± 2.67	< 0.0001
MMSE	28.7 ± 3.0	27.2 ± 2.7	19.2 ± 9.2	< 0.0001
MNA	12.2 ± 1.7	9.8 ± 2.8	7.7 ± 3.1	< 0.0001
LOS	8.2 ± 5.5	12.2 ± 10.2	15.7 ± 15.7	0.094

Unless otherwise noted, data are presented as means ± SD

*BMI* body mass index, *BADL* Basic Activities of Daily Living, *IADL* Instrumental Activities of Daily Living, *MMSE* Mini-Mental State Examination (corrected by age and education), *MNA* Mini Nutritional Assessment, *LOS* length of hospital stay

*χ^2^ = 6.723

**Table 4:** Linear regression analysis exploring HGS association with LOS controlling for multiple confounding factors (*n* = 108)

	*B*	*p*
Age	– 0.091	0.296
Gender	7.272	0.020
BMI	0.227	0.375
Frailty	0.633	0.669
TPS	7.444	0.002
MNA	– 0.692	0.128
HGS	– 0.343	0.020

Gender indicated as *F* = 0 e *M* = 1; *BMI* body mass index; frailty indicated as fit = 1 pre-frail = 2 and frail = 3; TPS type of surgery indicated as general surgery = 1, general oncological surgery = 2; MNA: Mini Nutritional Assessment

*HGS* handgrip strength

### Handgrip strength and length of hospital stay

**Fig. 1:**
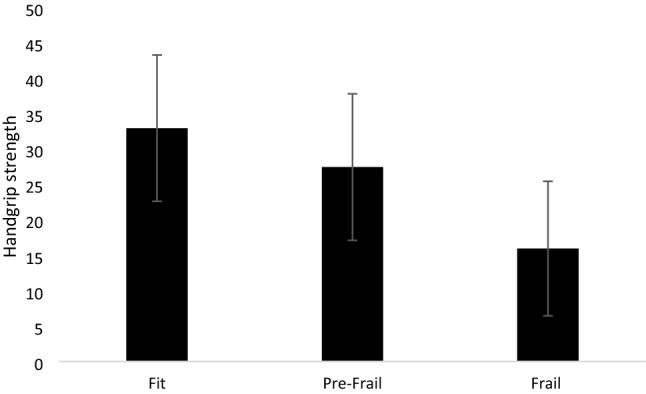
Handgrip strength (kg) in fit, pre-frail and frail subjects (*n* = 108; mean age 67.8 years old). Data are presented as means ± SD; Handgrip strength is expressed in kg. Fit: 32.99 ± 10.34; pre-frail: 27.49 ± 10.35; frail: 15.96 ± 9.52, *p* < 0.0001 by ANOVA

Handgrip strength significantly differed between fit, pre-frail and frail subjects (*p* < 0.001) as shown in Fig. 1. A simple correlation showed an inverse correlation between HGS and LOS (*r* = − 0.199; *p* = 0.040), even after adjustment for age and gender (*r* = − 0.220; *p* = 0.020). The independent effect of HGS on LOS was tested by a linear regression analysis controlling by multiple covariates (Table 4). HGS was significantly and inversely associated with LOS (*p* = 0.020) independent of age, gender, BMI, frailty status as a whole, type of surgery and MNA. Male gender and general oncological surgery also were significantly associated with LOS.

## Discussion

Our results show that: (1) the majority of population undergoing abdominal surgery is pre-frail or frail. (2) Handgrip strength significantly correlates with LOS independent of chronological age and gender. (3) HGS is significantly and inversely associated with LOS independent of chronological age, gender, BMI, frailty status, type of surgery, and nutritional status.

Recent evidence shows that frailty is a common condition in the surgical setting with significant postoperative implications. Frail surgical patients have higher rates of adverse health outcomes including prolonged hospital stay [27]. Defining surgical risk in this population can be difficult and the consideration of chronological age may be insufficient. Frequently, age-related changes in organs, tissues, and systems as a whole lead to the loss of functional and cognitive reserve, which may get out under a stressful condition, such as surgery. However, frailty is not easy to recognize as well as it is often misunderstood. Numerous tools, so far have been developed to measure frailty, but there is no standardized and validated method for assessment or screening in the peri-operative context. In our setting, we looked at frailty status independent of age according to Fried’s criteria, finding that in a population with a mean age of 67.8 years, 53.7% and 30.6% of patients were pre-frail or frail. These data are consistent with a recent meta-analysis [28] of nine observational studies including only older persons (over 65 years old), covering a wide range of upper and lower abdominal surgical conditions due to both benign and malignant conditions. The prevalence of pre-frail ranged between 31.3% and 45.8%, while frailty prevalence ranged between 10.4% and 37.0% [28]. Our study, instead, is the first to characterize frailty in a general surgery and elective setting with no chronological age limit (age range 19–93 years old), providing further evidence that frailty status could be independent from chronological age in such a setting.

Handgrip strength may be considered as a useful, easy-to-get and objective marker of frailty and already included in several frailty scoring systems, more time consuming, such as Fried’s classification [22]. Interestingly, HGS has been shown to independently predict adverse health outcomes and mortality in many older populations [29] and different clinical settings. To our knowledge, the evidence in the surgical field and, particularly, in the abdominal setting is poor. A previous study demonstrated that preoperative HGS may be considered a powerful predictor of postoperative pneumonia, LOS, institutionalization, and mortality after esophagectomy [30]. Another study also showed that low HGS was a significant risk factor for morbidity after gastric cancer surgery [31]. A systematic review [32] further showed that impaired preoperative HGS may be associated with poorer postoperative outcomes, including morbidity, LOS and mortality. The identification of patients at risk of prolonged hospital stay is a key element in the surgical setting considering that it may allow physicians to target appropriate timely interventions, to provide informed prognosis and to manage health-care resources effectively. In fact, LOS reflects the prognosis of the patient and it has been frequently used as an outcome of all changes in health status as a consequence of hospitalization [33]. Our study showed that HGS is significantly and inversely associated with LOS independent of chronological age, gender, BMI, frailty status as a whole, type of surgery, and nutritional status. This evidence strongly supports that HGS may provide a more useful single marker for LOS after adjustment for multiple covariates including frailty, nutritional status, and the type of surgery. Our data collectively show that muscle strength, as measured by handgrip dynamometry, is a strong predictor, together with male gender and oncological surgery, of LOS in a surgical setting. Making decisions based only on chronological age could be a failure.

### Strengths and limitations

 The strength of this study lies in its design with adequate sample size, and the use of HGS as an objective physical measurement. On the other hand, the patient cohort is from a single institution which represents a limitation. Moreover, because this was a retrospective study, selection bias could not be ruled out. Indeed, limited evidence suggests that frailty measures have predictive validity also in adult populations. Further research is needed to clarify the validity of measures across the adult age spectrum and explore the utility of measuring frailty in the surgical setting beyond age itself.

## Conclusions

The measurement of HGS is simple and inexpensive and provides objective powerful prognostic information for outcome after abdominal surgery. The simplicity of this measurement supports its usefulness as a tool to predict who will likely take longer to hospital discharge in surgical settings. Future research should focus on HGS and other physical and functional parameters (by a comprehensive geriatric assessment) and their associations with postoperative outcomes.

## Data Availability

The datasets used and/or analyzed during the current study will be available from the corresponding author on reasonable request.

## References

[1] Boccardi V, Marano L (2020). The geriatric surgery: the importance of frailty identification beyond chronological age. Geriatrics.

[2] St-Louis E, Sudarshan M, Al-Habboubi M (2016). The outcomes of the elderly in acute care general surgery. Eur J Trauma Emerg Surg.

[3] Makary MA, Segev DL, Pronovost PJ (2010). Frailty as a predictor of surgical outcomes in older patients. J Am Coll Surg.

[4] Han B, Li Q, Chen X (2019). Effects of the frailty phenotype on post-operative complications in older surgical patients: a systematic review and meta-analysis. BMC Geriatr.

[5] Kerr A, Syddall HE, Cooper C (2006). Does admission grip strength predict length of stay in hospitalised older patients?. Age Ageing.

[6] McNicholl T, Curtis L, Dubin JA (2020). Handgrip strength predicts length of stay and quality of life in and out of hospital. Clin Nutr.

[7] Kehler DS, Ferguson T, Stammers AN (2017). Prevalence of frailty in Canadians 18–79 years old in the Canadian Health Measures Survey. BMC Geriatr.

[8] Smart R, Carter B, McGovern J (2017). Frailty exists in younger adults admitted as surgical emergency leading to adverse outcomes. J Frailty Aging.

[9] Porto JM, Nakaishi APM, Cangussu-Oliveira LM (2019). Relationship between grip strength and global muscle strength in community-dwelling older people. Arch Gerontol Geriatr.

[10] White JV, Guenter P, Jensen G (2012). Consensus statement: academy of nutrition and dietetics and American society for parenteral and enteral nutrition. J Parenter Enter Nutr.

[11] Norman K, Stobäus N, Gonzalez MC (2011). Hand grip strength: Outcome predictor and marker of nutritional status. Clin Nutr.

[12] Mendes J, Azevedo A, Amaral TF (2014). Handgrip strength at admission and time to discharge in medical and surgical inpatients. J Parenter Enter Nutr.

[13] Syddall H, Cooper C, Martin F (2003). Is grip strength a useful single marker of frailty?. Age Ageing.

[14] Frederiksen H, Hjelmborg J, Mortensen J (2006). Age trajectories of grip strength: cross-sectional and longitudinal data among 8,342 danes aged 46 to 102. Ann Epidemiol.

[15] Budziareck MB, Pureza Duarte RR, Barbosa-Silva MCG (2008). Reference values and determinants for handgrip strength in healthy subjects. Clin Nutr.

[16] Mahalakshmi VN, Ananthakrishnan N, Kate V (2004). Handgrip strength and endurance as a predictor of postoperative morbidity in surgical patients: can it serve as a simple bedside test?. Int Surg.

[17] Guo C-B, Zhang W, Ma D-Q (1996). Hand grip strength: an indicator of nutritional state and the mix of postoperative complications in patients with oral and maxillofacial cancers. Br J Oral Maxillofac Surg.

[18] Chen C-H, Ho-Chang H-Z, Hung T-T (2011). Hand-grip strength is a simple and effective outcome predictor in esophageal cancer following esophagectomy with reconstruction: a prospective study. J Cardiothorac Surg.

[19] Ethun CG, Bilen MA, Jani AB (2017). Frailty and cancer: Implications for oncology surgery, medical oncology, and radiation oncology. CA Cancer J Clin.

[20] Handforth C, Clegg A, Young C (2015). The prevalence and outcomes of frailty in older cancer patients: a systematic review. Ann Oncol.

[21] Spiers GF, Kunonga TP, Hall A (2021). Measuring frailty in younger populations: a rapid review of evidence. BMJ Open.

[22] Fried LP, Tangen CM, Walston J (2001). Frailty in older adults: evidence for a phenotype. Journals Gerontol Ser A Biol Sci Med Sci.

[23] Folstein MF, Folstein SE, McHugh PR (1975). Mini-mental state. J Psychiatr Res.

[24] Katz S (1963). Studies of illness in the aged. JAMA.

[25] Graf C, Hartford Institute for Geriatric Nursing (2008). The Lawton instrumental activities of daily living (IADL) scale. Medsurg Nurs.

[26] Guigoz Y, Vellas B (1999) The Mini Nutritional Assessment (MNA) for Grading the Nutritional State of Elderly Patients: presentation of the MNA, History and Validation. In: Mini Nutritional Assessment (MNA): Research and Practice in the Elderly. Nutrition 15:116–122. 10.1016/s0899-9007(98)00171-3. 10.1159/000062967 11490593

[27] Richards SJG, Frizelle FA, Geddes JA (2018). Frailty in surgical patients. Int J Colorectal Dis.

[28] Hewitt J, Long S, Carter B (2018). The prevalence of frailty and its association with clinical outcomes in general surgery: a systematic review and meta-analysis. Age Ageing.

[29] Sasaki H, Kasagi F, Yamada M (2007). Grip strength predicts cause-specific mortality in middle-aged and elderly persons. Am J Med.

[30] Colcord ME, Benbow JH, Trufan S (2021). Preoperative muscle strength is a predictor of outcomes after esophagectomy. J Gastrointest Surg.

[31] Sato T, Aoyama T, Hayashi T (2016). Impact of preoperative hand grip strength on morbidity following gastric cancer surgery. Gastric Cancer.

[32] Sultan P, Hamilton MA, Ackland GL (2012). Preoperative muscle weakness as defined by handgrip strength and postoperative outcomes: a systematic review. BMC Anesthesiol.

[33] Brasel KJ (2007). Length of stay. Arch Surg.

